# An Optimized Self-Compensated Solution for Temperature and Strain Cross-Sensitivity in FBG Interrogators Based on Edge Filter

**DOI:** 10.3390/s21175828

**Published:** 2021-08-30

**Authors:** Mariana L. Silveira, Helder R. O. Rocha, Paulo F. C. Antunes, Paulo S. B. André, Marcelo E. V. Segatto, Anselmo Frizera, Camilo A. R. Díaz

**Affiliations:** 1 Telecommunications Laboratory LABTEL, Electrical Engineering Department, Federal University of Espírito Santo, Vitória 29075-910, Brazil; helder.rocha@ufes.br (H.R.O.R.); segatto@ele.ufes.br (M.E.V.S.); frizera@ieee.org (A.F.); c.rodriguez.2016@ieee.org (C.A.R.D.); 2 I3N & Physics Department, University of Aveiro, 3810-193 Aveiro, Portugal; pantunes@ua.pt; 3 Instituto de Telecomunicações, Campus Universitário de Santiago, 3810-193 Aveiro, Portugal; 4 Department of Electrical and Computer Engineering, Instituto de Telecomunicações, Instituto Superior Técnico, University of Lisbon, 1049-001 Lisbon, Portugal; paulo.andre@lx.it.pt

**Keywords:** fiber optical sensors, fiber Bragg gratings, edge filter, temperature cross-sensitivity, grey wolf optimization algorithm

## Abstract

Optical fiber sensors based on fiber Bragg gratings (FBGs) are prone to measurement errors if the cross-sensitivity between temperature and strain is not properly considered. This paper describes a self-compensated technique for canceling the undesired influence of temperature in strain measurement. An edge-filter-based interrogator is proposed and the central peaks of two FBGs (sensor and reference) are matched with the positive and negative slopes of a Fabry–Perot interferometer that acts as an optical filter. A tuning process performed by the grey wolf optimizer (GWO) algorithm is required to determine the optimal spectral characteristics of each FBG. The interrogation range is not compromised by the proposed technique, being determined by the spectral characteristics of the optical filter in accordance with the traditional edge-filtering interrogation. Simulations show that, by employing FBGs with optimal characteristics, temperature variations of 30 °C led to an average relative error of 3.4% for strain measurements up to 700μ*ϵ*. The proposed technique was experimentally tested under non-ideal conditions: two FBGs with spectral characteristics different from the optimized results were used. The temperature sensibility decreased by 50.8% as compared to a temperature uncompensated interrogation system based on an edge filter. The non-ideal experimental conditions were simulated and the maximum error between theoretical and experimental data was 5.79%, proving that the results from simulation and experimentation are compatible.

## 1. Introduction

Optical fiber sensing has been continuously evolving since its first observations in 1970 due to various advantages over conventional electronic sensing, such as immunity to electromagnetic interference, galvanic isolation, harsh environment suitability, no need for electrical power at the measuring point, compactness and multiplexing capability [1,2]. Notably, optical fiber sensors based on fiber Bragg gratings (FBGs) have gained special attention among the scientific and industrial communities. An FBG may be defined as a periodic modulation of the refractive index along the optical fiber’s core [3], which causes its backward-propagating spectrum to contain a peak centered at the so-called Bragg wavelength. The broad usability in sensing applications stems from its accurate sensitivity to axial strain and/or temperature variation, as the Bragg wavelength shifts under conditions of deformation and thermal changes. Therefore, by tracking the Bragg wavelength of an FBG, strain and temperature may be estimated and exploited to provide several measurands such as humidity, pressure, magnetic field, vibration, acceleration, liquid level detection and concentration of a specific soluble [4,5,6,7,8]. For these reasons, FBG-based sensors have been an attractive alternative for sensing applications in various fields, e.g., structural health, chemical industry, aerospace, robotics and medicine [9,10,11,12,13].

Different techniques may be used for interrogating FBG sensors. The most widespread approaches are based on optical filters [14,15,16] (e.g., edge filters, Fabry–Perot (FP) tunable filters and acousto-optic tunable filters), wavelength-swept lasers [17], interferometry (e.g., unbalanced Michelson and Mach–Zehnder (MZ) interferometers) [18], spectroscopy (e.g., Fourier transform spectrometer and linear-array detector spectrometer) [19,20], optical time-domain reflectometry (OTDR) [21], optical frequency-domain reflectometry (OFDR) [22], diffraction gratings and arrayed waveguide gratings [23]. All these techniques have been employed in the assessment of different measurands and each approach has its own advantages and drawbacks. Commonly, commercial interrogation systems rely on wavelength-swept lasers as they feature relatively high resolution, wavelength demodulation range and scanning speed [24]. However, these solutions are costly in comparison to alternative interrogation techniques. Systems based on optical spectrum analyzers (OSAs) are very accurate; nonetheless they are usually bulk (with poor portability) and expensive and have low acquisition frequency. Interferometry-based techniques are well-known for their high accuracy and sensitivity; however, some setups require optical devices, which increase the overall cost and size of the system, e.g., narrow-band optical sources, arrayed waveguide gratings and high-speed fiber stretchers [18,25]. Although edge-filter-based interrogators are sensible to optical source power fluctuations and usually exhibit a lower wavelength range, they are still often used as they are compact, portable, exhibit ease of signal processing and have high speed [15]. In contrast to interrogation systems based on wavelength scanning, they require lower acquisition rates and thus more inexpensive hardware [26]. In addition, some researches have pointed to inexpensive fabrication processes of edge filters based on Fabry–Perot interferometers (FPIs), e.g., the recycling of a standard single-mode fiber affected by the catastrophic fuse effect [27,28].

FBG sensors may deliver measurement errors if the cross-sensitivity between temperature and strain is not properly taken into account. With regard to strain assessment, a common approach to mitigate cross-sensitivity problems consists of employing a second FBG as a temperature sensor and removing the temperature influence via data processing through the coefficient matrix [29]. However, this technique requires wavelength information, which is often provided by costly and complex interrogation systems. To overcome this disadvantage, Zhou et al. [30] developed a similar approach that applies the coefficient matrix directly to the optical power information. Nonetheless, this approach employs an extra edge filter that acts as the reference element, instead of a second FBG.

Furthermore, techniques that do not rely on data processing were developed for canceling temperature sensitivity in strain interrogators based on an edge filter. Wu et al. [31] employed a second FBG as an edge filter and submitted it to equal environmental conditions as compared with the FBG sensor. Both sensing and edge filtering elements exhibited similar wavelength shifts, minimizing the error driven by cross-sensitivity between temperature and strain. The main disadvantage of this approach is the non-linearity induced by the FBG-based edge filter. An analogous working principle was explored by Miao et al. [32]. They employed a tilted FBG as an edge filter after immersing it in an index-matching gel to acquire a smoother response. Nevertheless, the refractive index of the gel may change over time due to environmental exposure, which would lead to future errors. In the method proposed by Ghosh et al. [33], the peak of a continuous-wave laser source is matched with the ascendant and descendant slopes of the backward-propagating spectrum of the sensing and reference FBGs. As temperature changes, one FBG provokes a decrease in optical power, whereas the other causes an increase by a similar amount, minimizing temperature sensitivity. However, further studies for determining the ideal spectral tuning were not undertaken, compromising the adaptability to systems that employ different optical sources and FBGs.

This paper presents a cost-effective solution for the cross-sensitivity between strain and temperature in FBG strain interrogators based on an edge filter. A second FBG is employed as a reference element and exposed to the same temperature changes as the strain sensing element. The proposed approach consists of matching the Bragg wavelengths of the sensing and reference FBGs with two opposite (negative and positive) slopes of the edge filter’s spectrum, minimizing optical power changes caused by temperature oscillations: one FBG’s convoluted signal produces a decrease in optical power, whereas the other produces an increase in optical power by a similar amount. To determine the ideal spectral tuning or, in other words, define the best spectral characteristics of the FBGs, the grey wolf optimization (GWO) algorithm is applied. The remainder of this paper is organized as follows. Section 2 presents a self-compensated solution for temperature and strain cross-sensitivity and the FBG tuning process. Section 3 depicts the results obtained from simulation and experiments. Finally, the conclusions are drawn in Section 4.

## 2. Materials and Methods

In addition to an edge filter, the proposed interrogation system contains two FBGs acting as reference ($FBG_{1}$) and sensing ($FBG_{2}$) elements. The sensor is exposed to axial deformation and temperature variations, whereas the reference is strain-free and experiences the same environmental conditions as the sensor. In order to minimize the optical power fluctuations provoked by external temperature changes, the Bragg wavelengths of both FBGs ought to match with two opposite slopes of the edge filter’s spectrum, as depicted by the following analysis.

### 2.1. Self-Compensated Technique: Working Principle

Assuming that a broadband optical source with power density equal to 1 is employed and that the bandwidths of the reflected spectra of $FBG_{1}$ and $FBG_{2}$ do not overlap each other, the overall optical power *P* that arrives at the photodetector may be defined as,

(1) $$P=r_{0}\int T_{F}\left(\lambda \right)\left(R_{1}\left(\lambda \right)+R_{2}\left(\lambda \right)\right)d\lambda$$

where $r_{0}$ is the photodetector’s responsiveness, λ is the wavelength, $T_{F}$ is the spectrum of an FPI acting as an edge filter and $R_{1}$ and $R_{2}$ are the backward-propagating spectra of $FBG_{1}$ and $FBG_{2}$, respectively. Equation (1) can be rewritten as shown below and therefore the overall optical power may be interpreted as the sum of the optical powers delivered by the convolution between each FBG and the edge filter’s signal.

(2) $$P=r_{0}\left(\int T_{F}\left(\lambda \right)R_{1}\left(\lambda \right)d\lambda +\int T_{F}\left(\lambda \right)R_{2}\left(\lambda \right)d\lambda \right)$$

(3) $$P=r_{0}\left(P_{1}\left(\lambda \right)+P_{2}\left(\lambda \right)\right)$$

Here, $P_{1}$ and $P_{2}$ represent the optical powers exclusively related to $FBG_{1}$ and $FBG_{2}$, respectively. The difference in optical power ΔP arising from two conditions in which the temperature varies from $T_{1}$ to $T_{2}$ is given by,

(4) $$\Delta P=r_{0}\left(P_{1}^{T2}\left(\lambda \right)-P_{1}^{T1}\left(\lambda \right)+P_{2}^{T2}\left(\lambda \right)-P_{2}^{T1}\left(\lambda \right)\right)$$

**Figure 1:**
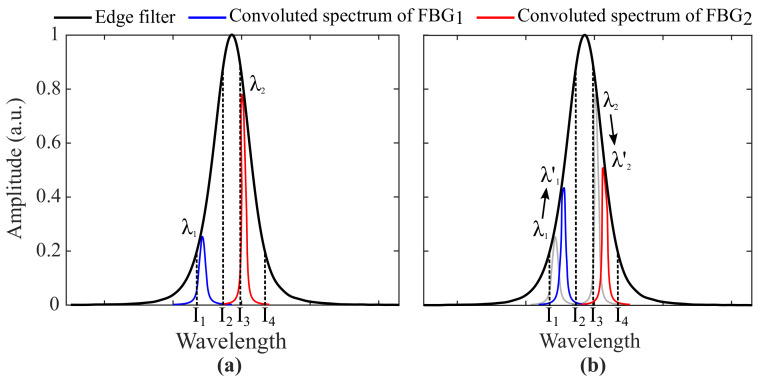
Working principles of the proposed self-compensated solution for temperature and strain cross-sensitivity. Two FBGs are exposed to different temperatures (**a**) $T_{1}$ and (**b**) $T_{2}$, where $T_{2}>T_{1}$. Spectral redshifts change the Bragg wavelengths from $\lambda_{1}$ and $\lambda_{2}$ to $\lambda_{1}^{\prime }$ and $\lambda_{2}^{\prime }$. The convolution between the FBGs’ and the edge filter’s spectra lead to an increase in optical power by means of $FBG_{1}$ and a decrease by means of $FBG_{2}$.

Considering that the Bragg wavelengths of $FBG_{1}$ ($\lambda_{1}$) and $FBG_{2}$ ($\lambda_{2}$) are contained within the intervals $I_{1}<\lambda_{1}<I_{2}$ and $I_{3}<\lambda_{2}<I_{4}$ (limits of the interrogation range), and that the edge filter behaves as a monotonic function with positive slope along the interval ($I_{1},I_{2}$) and negative slope along ($I_{3},I_{4}$) as exhibited in Figure 1a, it can be noted that external temperature variations lead to shifts in $\lambda_{1}$ and $\lambda_{2}$ causing linear and positive optical power variations via $FBG_{1}$ and linear and negative variations via $FBG_{2}$ (Figure 1b). As can be derived from Equation (4), the overall optical power P(λ) is not affected by temperature if shifts in the Bragg wavelength $\lambda_{1}$ provoke an increase in optical power and shifts in $\lambda_{2}$ provoke a decrease by the same amount.

In the proposed technique, the minimization of temperature cross-sensitivity occurs dynamically within the optical components, without the need to perform any posterior data processing. For this reason, the system herein presented is considered to be self-compensated. The strain data can be obtained just as in any conventional edge-filter-based interrogation system, e.g., by means of an optical power meter and a strain characterization curve. As temperature variations do not interfere in the overall optical power, this system exclusively measures strain. However, arrangements in the proposed setup may be performed so that temperature measurements are also provided. This is further discussed in Section 2.5.

The proposed method has the benefits of a conventional edge-filter-based interrogator. In comparison with the interrogation systems based on wavelength-swept lasers, optical spectrum analyzers, spectrometers and OFDR and OTDR techniques, the edge-filter-based schemes have compact size, light weight, ease of signal processing, reduced number of optical components and more inexpensive hardware [15,26]. In addition, the proposed method enables overcoming the cross-sensitivity between temperature and strain, one of the major drawbacks of some FBG interrogators based on an optical filter.

### 2.2. Mathematical Models

The GWO algorithm is used to determine the optimal spectral characteristics of $FBG_{1}$ and $FBG_{2}$ and minimize optical power fluctuations caused by temperature changes. For this reason, the mathematical models of the system’s optical elements are required. Equation (5) describes the backward-propagating spectrum of a uniform FBG ($R_{x},x=1,2$) as a function of its grating length ($L_{x}$) and wavelength (λ) [34].

(5) $$R_{x}\left(\lambda ,L_{x}\right)=\frac{k^{2}\sinh\left(L_{x}\gamma \right)}{{\hat{\sigma }}^{2}\sinh^{2}\left(L_{x}\gamma \right)+\gamma^{2}\cosh^{2}\left(L_{x}\gamma \right)},$$

where *k* is the ‘AC’ coupling coefficient, ${\hat{\sigma }}^{2}$ is the general ‘DC’ self-coupling coefficient, and $\gamma =\sqrt{|k^{2}-{\hat{\sigma }}^{2}|}$. The remaining parameters are given by:

(6) $$k=\frac{\pi }{\lambda }\upsilon_{n}{\bar{\delta n}}_{\mathrm{eff}},$$

(7) $$\hat{\sigma }=\zeta_{x}+\sigma ,$$

(8) $$\sigma =\frac{2\pi }{\lambda }\upsilon_{n}{\bar{\delta n}}_{\mathrm{eff}},$$

(9) $$\zeta_{x}=2\pi n_{\mathrm{eff}}\left(\frac{1}{\lambda }-\frac{1}{\lambda_{x}}\right),$$

where $n_{\mathrm{eff}}$ is the effective refractive index, ${\bar{\delta n}}_{\mathrm{eff}}$ is the average refractive index perturbation over a grating period (’DC’ index changes) and $\upsilon_{n}$ is the fringe visibility of the index change. The Bragg wavelength $\lambda_{x}\left(x=1,2\right)$ is given by:

(10) $$\lambda_{x}=\lambda_{x_{0}}\left[1+\left(1-p_{e}\right)\epsilon +\left(\alpha +\xi \right)\Delta T\right],$$

where $\lambda_{x_{0}}$ is the designed Bragg wavelength, $p_{e}$ is the effective photo-elastic coefficient, ϵ is the transverse strain, α is the thermal expansion coefficient of the optical fiber material, ξ is the thermo-optic coefficient and ΔT is the temperature variation.

Additionally, Equations (11) and (12) describe the forward-propagating spectrum of an FPI as a function of the wavelength.

(11) $$T_{FPI}\left(\lambda \right)=\frac{{\left(1-r\right)}^{2}}{{\left(1-r\right)}^{2}+4r\sin^{2}\left(\delta /2\right)},$$

(12) $$\delta =\frac{4\pi nl\cos\theta }{\lambda },$$

where *r* is the mirror’s reflectance, *n* is the reflective index of the micro-cavity’s material, θ is the internal angle of incidence of the beam into the micro-cavity and *l* is the reflector’s distance.

The parameter setting is outlined in Table 1 and conforms to Díaz et al. [14].

### 2.3. Grey Wolf Optimizer

Mirjalili et al. [35] proposed a meta-heuristic optimization technique named the grey wolf optimizer, since it is inspired by the behavior and hierarchical organization of grey wolves. In this technique, the population of search agents is symbolized as wolves and the fittest solution is symbolized as the prey. The most reliable candidates are called alpha, beta and delta in accordance with the nomenclature assigned to the three highest ranks within the grey wolf hierarchy. The information of alpha, beta and delta is used to update all the elements of the population at each iteration by means of a mathematical model inspired by the encircling behavior of grey wolves during hunting. The algorithm’s final outcome is the position of the best candidate, namely the alpha search agent. One advantage of the GWO is to have multiple candidate solutions that share information about the search space. In addition, this technique is simple, easy to implement, has high local optima avoidance and fewer parameters for adjustments [35].

The main mathematical equations of the GWO model are given by:

(13) $$\bar{X}\left(t+1\right)=\frac{{\bar{X}}_{1}+{\bar{X}}_{2}+{\bar{X}}_{3}}{3}$$

(14) $${\bar{X}}_{1}={\bar{X}}_{\alpha }-{\bar{A}}_{1}\cdot {\bar{D}}_{\alpha },{\bar{X}}_{2}={\bar{X}}_{\beta }-{\bar{A}}_{2}\cdot {\bar{D}}_{\beta },{\bar{X}}_{3}={\bar{X}}_{\delta }-{\bar{A}}_{3}\cdot {\bar{D}}_{\delta }$$

(15) $${\bar{D}}_{\alpha }=|{\bar{C}}_{1}\cdot {\bar{X}}_{\alpha }-\bar{X}|,{\bar{D}}_{\beta }=|{\bar{C}}_{2}\cdot {\bar{X}}_{\beta }-\bar{X}|,{\bar{D}}_{\delta }=\left|{\bar{C}}_{3}\cdot {\bar{X}}_{\delta }-\bar{X}\right|,$$

where $\bar{X}$ represents the position of a search agent as a function of the iteration *t* and ${\bar{X}}_{\alpha }$, ${\bar{X}}_{\beta }$ and ${\bar{X}}_{\delta }$ represent the positions of the alpha, beta and delta search agents, respectively. The coefficient vectors $A_{x}$ and $C_{x}\left(x=1,2,3\right)$ are calculated as follows:

(16) $${\bar{A}}_{x}=b\left(2a\cdot {\bar{r}}_{1}-a\right),$$

(17) $${\bar{C}}_{x}=2\cdot {\bar{r}}_{2},$$

where *a* is linearly decreased from 2 to 0 over the course of iterations and ${\bar{r}}_{1}$ and ${\bar{r}}_{2}$ are random vectors whose elements are within the interval (0, 1). The constant *b* does not exist in the original mathematical model; however, it is necessary in this work to reduce cases in which the positions of the search agents $\bar{X}$ are set outside the search space (0>b>1). This adjustment is due to the narrow search space of the proposed problem, which is related to the edge filter’s transition band.

### 2.4. FBG Tuning via GWO

**Figure 2:**
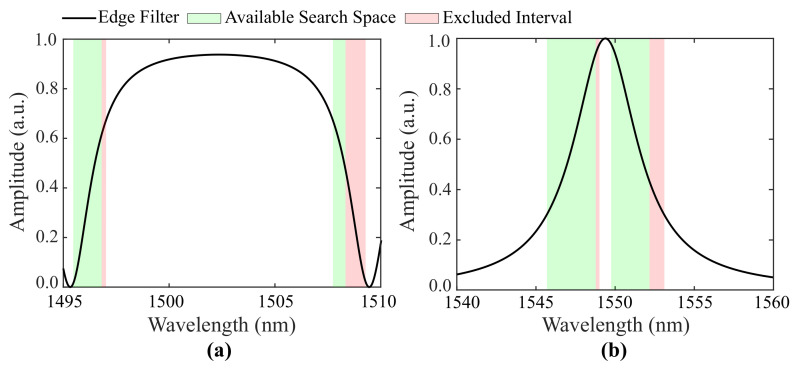
Examples of the search space of $\lambda_{1_{0}}$ and $\lambda_{2_{0}}$ for (**a**) a backward-propagation spectrum and (**b**) the forward-propagation spectrum of an FPI.

The inputs required by GWO are the number of search agents, the number of iterations and the search space of each element of $\bar{X}$. In this work, $\bar{X}$ is a four-dimensional column-array whose elements represent the designed Bragg wavelength $\lambda_{1_{0}}$ of $FBG_{1}$, the designed Bragg wavelength $\lambda_{2_{0}}$ of $FBG_{2}$, the grating length $L_{1}$ of $FBG_{1}$ and the grating length $L_{2}$ of $FBG_{2}$. Regarding the search spaces of $L_{1}$ and $L_{2}$, these are recommended as (3,10) mm, since this interval includes the values most commonly found in the literature [14]. It is worth noting that the FBG’s length is related to the spectral visibility and bandwidth. The search spaces of $\lambda_{1_{0}}$ and $\lambda_{2_{0}}$ are the most linear regions of the edge filter’s transition bands with positive and negative slope, respectively. To prevent the FBGs from exceeding these linear regions, the search space of $FBG_{2}$ is subtracted by $s_{\epsilon }\cdot \epsilon_{max}$, where $s_{\epsilon }$ represents the wavelength shift per strain and $\epsilon_{max}$ represents the maximum strain applied over the sensor. Shifts caused by temperature are also taken into account, leading to a decrease in the search space of both FBGs by $s_{T}\cdot T_{max}$, where $s_{T}$ represents the wavelength shift per temperature and $T_{max}$ represents the maximum temperature at which the interrogator operates. Figure 2 shows two examples of the search space of $\lambda_{1_{0}}$ and $\lambda_{2_{0}}$ for different edge filters, as well as their excluded intervals.

During each iteration of GWO, a simulation of the system’s response for a strain-free condition with variable temperature is performed. The temperature (*T*) varies linearly from $T_{min}$ to $T_{max}$ in steps of $\delta_{T}$ and, at each step, *P* is calculated by means of Equations (1), (5) and (11). Thus, the optical power curve versus temperature P(T) is obtained and the cost function (which GWO aims to minimize) is considered to be the difference between the maximum and minimum values of $P_{norm}\left(T\right)$ multiplied by its standard deviation σ (Equation (18)), where $P_{norm}\left(T\right)=P\left(T\right)-P\left(T=T_{min}\right)$.

(18) $$CostFunction=(max\left(P_{norm}\left(T\right)\right)-min\left(P_{norm}\left(T\right)\right)\cdot \sigma (P_{norm})$$

Table 2 lists the parameter settings. The number of iterations and the number of search agents are determined based on the common literature [36,37]. The remaining parameters are set based on the spectral characteristics of the edge filter chosen for experimentation and based on empirical analysis during small simulations.

### 2.5. Practical Experimentation: A Strain-Free Experiment for Temperature Sensitivity Analysis in Non-Ideal Conditions

Many times in practical application, experimentation requires resources readily available at the research laboratory. For this reason, an experiment is performed under non-ideal conditions. Two FBGs whose Bragg wavelengths are slightly different from the optimized $\lambda_{1}{}_{0}$ and $\lambda_{2}{}_{0}$ are chosen to integrate the experimental setup. In addition, their grating lengths do not match with the outputs $L_{1}$ and $L_{2}$ of GWO and their spectral side lobes are not consistent with the assumptions made in Equation (5). Therefore, we consider this condition non-ideal. However, it is worth noting that FBGs with low-amplitude side lobes or apodization can be employed to sustain the assumptions of Equation (5) [38].

**Figure 3:**
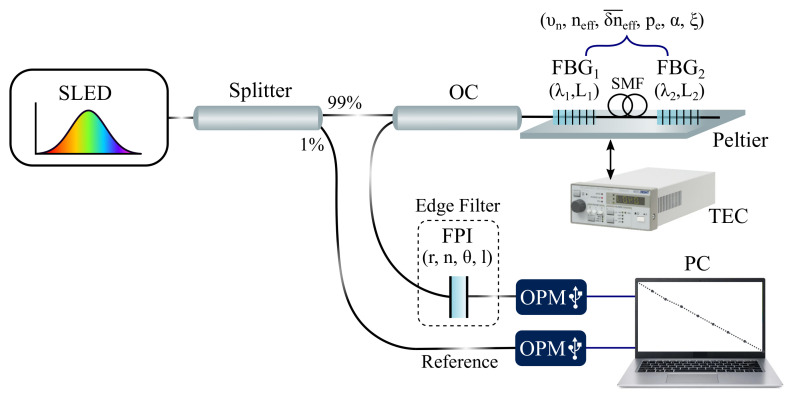
Schematic presentation of the proposed system. The parameters listed in Table 1 are exhibited near the optical components with which they are associated: r,n,θ,l are related to the FPI; $\lambda_{1},L_{1}$ and $\lambda_{2},L_{2}$ are related to $FBG_{1}$ and $FBG_{2}$, respectively; and $\upsilon_{n},n_{\mathrm{eff}},{\bar{\delta n}}_{\mathrm{eff}},p_{e},\alpha ,\xi$ are herein considered common for both FBGs.

The system depicted in Figure 3 is proposed for assessing the temperature sensitivity in a strain-free non-ideal condition. To provide a better understanding of the mathematical models, the parameters listed in Table 1 are exhibited in Figure 3 near the optical component with which they are related. In the proposed setup, the light emitted by a super-luminescent diode centered at 1550 nm with a bandwidth of 60 nm (SLED, DL-BP1-1501A, Ibsen Photonics, Farum, Denmark) is launched into an optical circulator (OC, 6015-3-APC, Thorlabs, Newton, NJ, USA) through a 99/1 optical splitter. The overall launched power is about 10 mW, of which 1% is monitored through an optical power meter (OPM, OPM5-4D, AFL, San Leandro, CA, USA) in order to identify light source fluctuations. The remaining source power is launched into two multiplexed FBGs ($FBG_{1}$ and $FBG_{2}$) inscribed in single-mode optical fibers (SMFs) with central Bragg wavelengths of 1547.6 nm and 1550.0 nm. A segment of SMF is spliced between the FBGs to enable their spatial detachment. To prevent the strain from being transferred to the reference, the sensor element may be fixed to the monitored surface by its extremities and the reference may be left loose within its protective material. Moreover, if the application requires the FBGs to be multiplexed in different optical fiber segments, a 50/50 optical splitter may be added after port 2 of the OC and each FBG may be connected to an output arm [29]. The FBGs’ backward-propagating signal is launched into an optical band-pass filter (TB1500B, JDS Fitel, USA) through port 3 of the OC. This optical filter consists of an FPI whose transmission spectrum is centered at 1549.4 nm with 4.825 nm of full-width at half-maximum (FWHM) and more than 35 dB of visibility. The optical power that results from the convolution between the sensors’ and filter’s optical signal is acquired by a second OPM (OPM5-4D, AFL, San Leandro, CA, USA) in the acquisition stage. The output of this OPM is subtracted from the derivative of the signal captured in the splitter’s 1% arm to cancel source fluctuations. The data are acquired with a sampling frequency of 5 Hz and transmitted via USB to a computer (PC) for data processing.

During experimentation, the FBGs are positioned on a Peltier plate whose initial temperature is 15 °C. A temperature controller (TEC, TED200C, Thorlabs, Newton, NJ, USA) is used to set the desired temperature and keep it constant with a temperature resolution of 0.01 °C and stability lower than 0.002 °C according to the user’s manual. The temperature is raised in steps of 5 °C until the maximum of 40 °C is achieved. Then, temperature is decreased back to 15 °C in equal steps. At each step, the measurements of both OPMs are collected for approximately five minutes. Finally, this non-ideal experimental condition is also simulated to prove that the experimental and theoretical results are compatible.

Considering that ideal conditions are guaranteed or, in order words, the spectral characteristics of the FBGs are consistent with the results of the optimizer, the system presented in Figure 3 is proposed as a tool for strain assessment. One possible solution to additionally provide temperature measurements consists of connecting a 1 × 2 optical splitter to port 3 of the OC and an extra edge filter to one of the splitter’s arms [30]. The transition band of this filter must match the central Bragg wavelength of the reference FBG in order to modulate only its wavelength shifts. In this approach, the other arm of the optical splitter must be connected to the FPI and its following components, as exhibited in Figure 3. The temperature information can be extracted as optical power by connecting the extra edge filter to a third OPM.

## 3. Results and Discussion

### 3.1. FBG Tuning via GWO

Figure 2b exhibits the spectrum of the edge filter calculated through Equation (11) and Table 1 for FBG tuning. It is worth noting that this signal must replicate the spectral characteristics of the optical filter chosen to integrate the interrogation system. In our case, it replicates the spectrum provided by the band-pass optical filter described in Section 2.5. Due to the spectral characteristics of our filter, the interrogation ranges are set from 15 °C to 45 °C and from 0μϵ to 700μϵ. However, different values can be achieved by employing different edge filters with broader or narrower transition bands. It is important to emphasize that the interrogation range is not determined by the technique herein proposed, but by the spectral characteristics of the chosen optical filter.

The search spaces of $\lambda_{1_{0}}$ and $\lambda_{2_{0}}$ are represented in Figure 2b, as well as the regions excluded due to temperature and strain shifts. As can be seen, the width of the search space depends on the width of the filter’s transition band. For this particular case, the bounds of the search space are (1545.7,1548.9) nm and (1549.6,1552.2) nm for $\lambda_{1_{0}}$ and $\lambda_{2_{0}}$, respectively.

**Figure 4:**
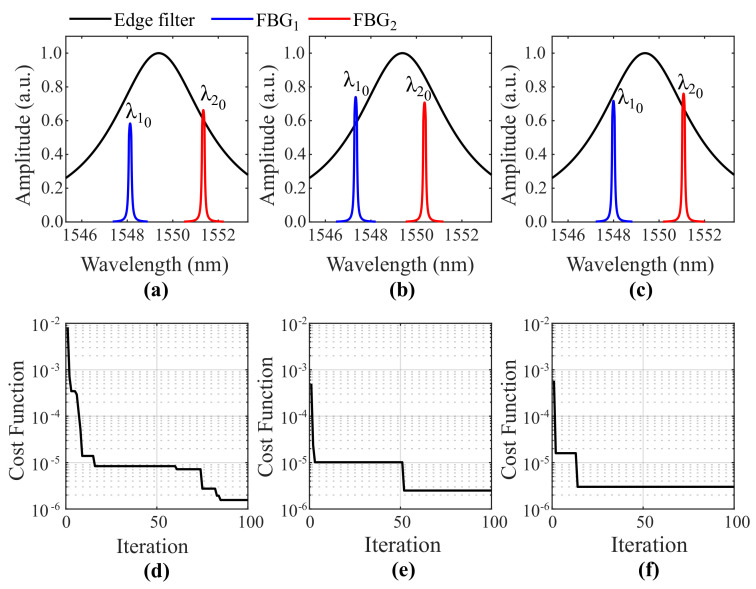
The three best results obtained after ten executions of the GWO algorithm: spectral information of the (**a**) first, (**b**) second and (**c**) third best alpha search agents; and the cost curve versus iteration that led to the (**d**) first, (**e**) second and (**f**) third best results.

As GWO is a meta-heuristic optimizer whose models contain random parameters, each execution generates slightly different outputs. Figure 4 depicts the three best results obtained after ten executions of the GWO algorithm or, in other words, the three alpha search agents that achieved the smallest cost functions, from the best (left) to the third best result (right). Their spectral information is shown in Figure 4a–c. In addition, the cost curve versus iteration related to each result is exhibited below in Figure 4d–f. As can be seen, GWO was capable of minimizing the cost function to values below $3.01\times 10^{-6}$ in all executions. Since different designed Bragg wavelengths were obtained in each execution, it is possible to conclude that the optimizer algorithm is not tied to a specific phase mask (required by some FBG inscription processes), which offers the proposed approach high flexibility for real implementation.

**Figure 5:**
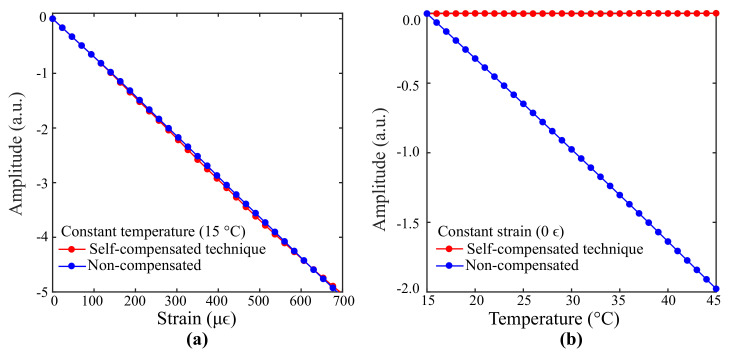
Comparison between the responses of an interrogation system with the proposed technique and a non-compensated interrogation system for (**a**) linear strain and (**b**) linear temperature variations.

Simulation analyses are now carried out for the 1st best alpha search agent ($\lambda_{1_{0}}=1547.22$ nm, $\lambda_{2_{0}}=1550.25$ nm, $L_{1}=6.37$ mm, $L_{2}=6.04$ mm). Firstly, the proposed technique is compared to a temperature non-compensated interrogation system that employs the edge filter whose parameters are listed in Table 2 (Figure 2b) and $FBG_{2}$ ($\lambda_{2_{0}}=1550.25$ nm, $L_{2}=6.04$) as the sensor element. The ideal case, in which temperature is maintained constant (*T* = 15 °C) while the systems are subjected to variable strain, is shown in Figure 5a. The maximum relative error between the optical power curves is 1.10% and the strain sensitivities of both systems differ by 0.11%. Therefore, it can be concluded that the proposed technique does not prejudice the sensor sensitivity or affect the measurements of a temperature non-compensated interrogation system based on an edge filter. The system responses for different temperatures and zero strain are exhibited in Figure 5b. The optical power curve delivered by the proposed technique is represented by a straight line with standard deviation equal to $8.12\times 10^{-4}$. However, the non-compensated system presents significant optical power variations.

**Figure 6:**
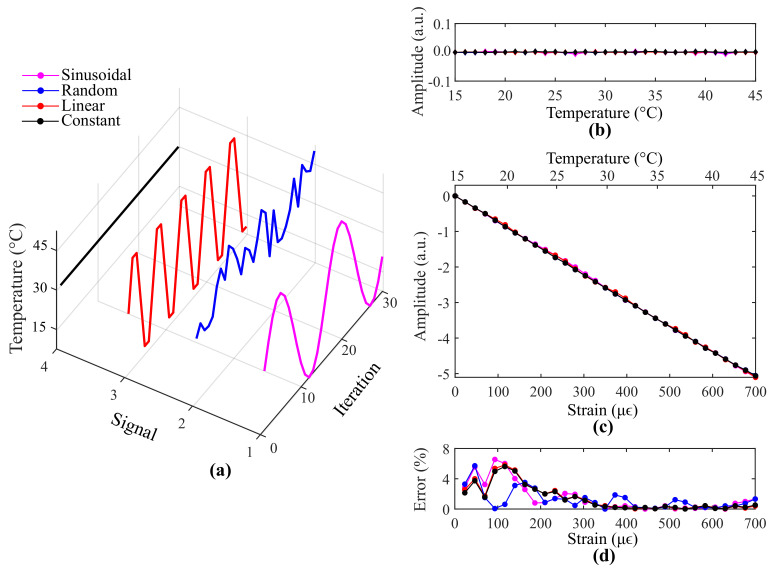
Results from the proposed technique for (**a**) different temperature signals in a (**b**) strain-free and (**c**) variable strain condition. The errors associated with the variable strain condition are shown in (**d**).

Different temperature behaviors over time are also analyzed. Temperature signals with sinusoidal, random, linear and constant characteristics are generated to investigate the responses delivered by the proposed technique. As exhibited in Figure 6a, the sinusoidal and linear signals vary from 15 °C to 45 °C, whereas the random signal varies from 30 °C to 45 °C. The constant signal was maintained at 30 °C. The system responses for a strain-free condition are shown in Figure 6b. As can be seen, the optical power curves are represented as straight lines. The maximum standard deviation is $2.1\times 10^{-3}$ and occurs for the optical power curve related to the sinusoidal signal. Figure 6c depicts the system responses for a variable strain condition. Their relative errors to the non-compensated response of Figure 5a are exhibited in Figure 6d. The maximum relative error is 6.51% and the average relative error considering all curves is 3.4%. Therefore, it can be concluded that the nature of the temperature signal does not interfere significantly in the system response. Finally, we consider that the self-compensated solution for strain and temperature cross-sensitivity herein presented is a promising tool for instantly canceling the effect of temperature on strain measurements.

### 3.2. Practical Experimentation: A Strain-Free Experiment for Temperature Sensitivity Analysis in Non-Ideal Conditions

**Figure 7:**
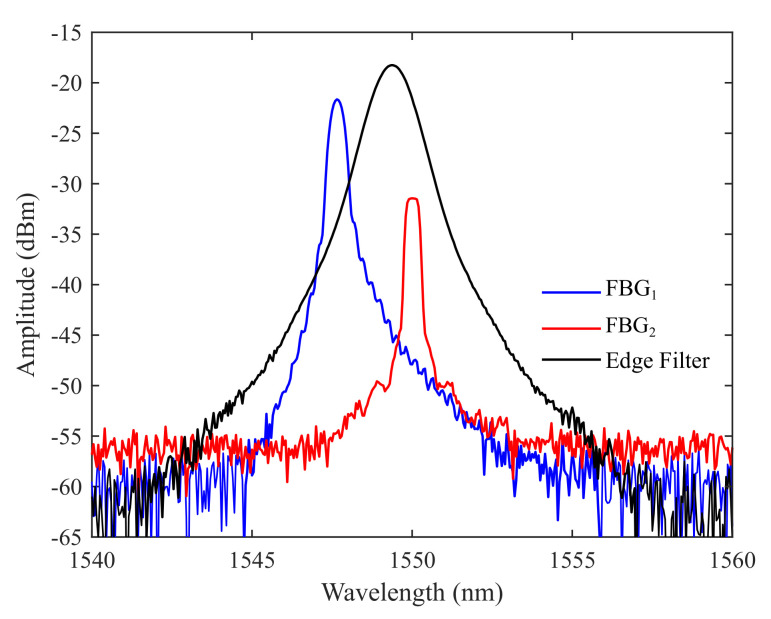
The spectra of the FBGs and the edge filter.

Figure 7 exhibits the spectra of the edge filter and FBGs ($FBG_{1}$ and $FBG_{2}$) chosen for experimentation. The spectral data were acquired by an OSA (MS9740B, Anritsu, Japan) with 30 pm resolution.

**Figure 8:**
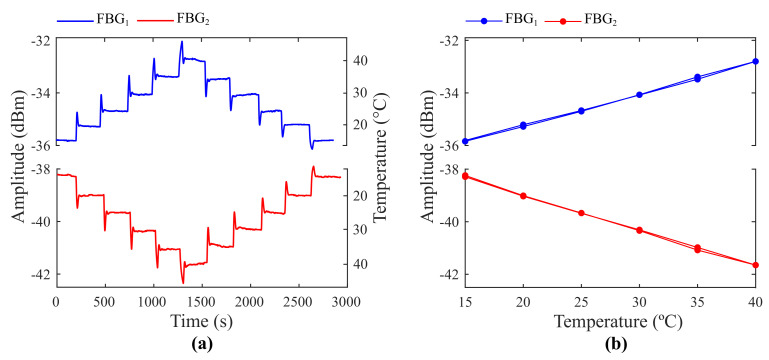
Optical power curves (**a**) versus time and (**b**) versus temperature for $FBG_{1}$ and $FBG_{2}$ in a non-multiplexed configuration with zero strain.

The optical power delivered by the convolution between the edge filter and FBG signals was acquired for different temperatures and constant strain (ϵ=0). Figure 8a shows the optical power curve versus time obtained when $FBG_{1}$ and $FBG_{2}$ were not multiplexed and, therefore, used separately in the setup exhibited in Figure 3. Source fluctuations were mitigated by subtracting the acquired signals by the source reference. Their underdamped behavior stems from the temperature controller of the Peltier plate, which provides temperature adjustments based on a PID controller. Hysteresis and optical power fluctuations are due to temperature errors associated with the control system. Figure 8b shows the system’s responsiveness to temperature for a non-multiplexed configuration. As can be seen, both FBGs delivered fairly linear results ($R^{2}=0.99$ for both $FBG_{1}$ and $FBG_{2}$) with low hysteresis (maximum hysteresis of −1.5% and 2.96% for $FBG_{1}$ and $FBG_{2}$, respectively). The estimated sensitivities were 0.12 dBm/°C and −0.14 dBm/°C for $FBG_{1}$ and $FBG_{2}$, respectively.

**Figure 9:**
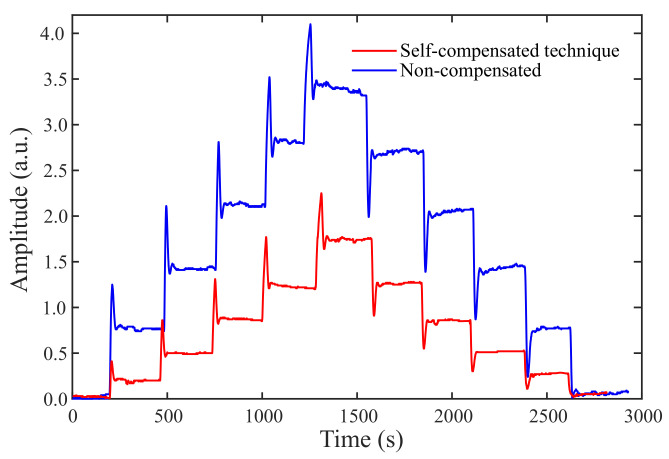
Optical power curves versus time for an interrogation system with the proposed technique and a non-compensated system considering linear temperature variations and zero strain.

The experimental results of the proposed technique for a non-ideal condition are exhibited in Figure 9, as well as the optical power curve delivered by the sensor ($FBG_{2}$) with no technique to mitigate the temperature influence. To ease comparison, the relative absolute values of both signals are illustrated. As can be seen, the temperature sensibility decreased from 0.14 dBm/°C to 0.07 dBm (a decrease of 50.8%), proving that the proposed technique has potential to minimize temperature cross-sensitivity.

**Figure 10:**
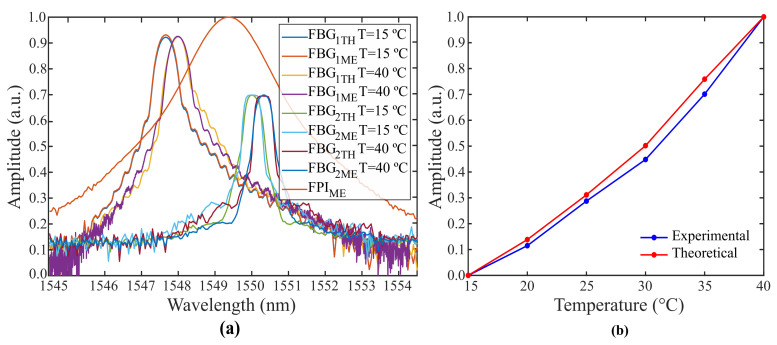
Comparison between theoretical and experimental data: (**a**) simulated and experimentally acquired spectra for *T* = 15 °C and *T* = 40°C and (**b**) simulated and experimental optical power curve versus temperature.

To contrast experimental and theoretical information, a simulation of the non-ideal experimental conditions was performed. The coupled-mode theory was applied to reproduce the FBG’s sidelobes and warrant a reliable copy of both spectra [39]. In addition, noise was added. The multiplexed signal of $FBG_{1}$ and $FBG_{2}$ was represented as the envelope between the FBG’s spectrum. Regarding the FPI, the measured spectrum was normalized and replicated in simulation. Figure 10a depicts the measured (ME) and theoretical (TH) spectra of $FBG_{1}$ and $FBG_{2}$ for *T* = 15 °C and *T* = 40 °C in a strain-free condition. The system’s responsiveness to temperature is shown in Figure 10b. The maximum absolute error between theoretical and experimental data is 5.79%. Due to this congruence, it is expected that, when FBGs with optimal spectral characteristics are integrated to the system, optical power fluctuations up to 30 °C will not have significant effect on strain measurements within the interrogation range of 700μϵ, as verified in the simulations. In addition, as can be noted in Figure 10b, the optical power curves exhibit nonlinear behavior although $FBG_{1}$ and $FBG_{2}$ presented linear responses when analyzed separately (Figure 8). This can be justified by the significant overlapping area between their reflected spectra. To prevent this, FBGs with low-amplitude side lobes or apodization must be employed along with the proposed technique.

## 4. Conclusions

This paper has presented a self-compensated solution for cross-sensitivity between temperature and strain in FBG interrogators based on an edge filter. Its working principle consists of matching two FBGs acting as reference and sensor elements at the ascending and descending slopes of an FPI employed as an optical filter. A tuning process performed by the grey wolf optimizer (GWO) algorithm is required to determine the optimal spectral characteristics of the FBGs. A constant was added to the mathematical model of GWO for adapting the algorithm’s search space to the narrow transition band of the edge filter. The responses of the proposed technique to different temperature signals with sinusoidal, random, linear and constant characteristics were analyzed via simulation. Under a strain-free condition, the optical power was represented as a straight line with a maximum standard deviation of $2.1\times 10^{-3}$. For a variable strain condition, temperature variations up to 30 °C led to an average relative error in strain measurements of 3.4%. Therefore, it can be concluded that the proposed technique is not affected by the nature of the temperature signal. In addition, it has been shown that the proposed technique does not influence the interrogation range or the system sensitivity to strain when compared to the traditional edge-filtering interrogation scheme. As practical experimentation sometimes requires resources that are readily available at the research laboratory, an experiment was performed under non-ideal conditions: the proposed technique was applied with two FBGs whose spectral characteristics are different from the GWO results. The temperature sensibility decreased by 50.8% in comparison with a temperature uncompensated interrogation system based on an edge filter. These non-ideal experimental conditions were simulated and the maximum error between theoretical and experimental data was 5.79%, proving that the results from simulation and experimentation are compatible. To ensure that the conditions of the proposed mathematical model remain valid, FBGs with low-amplitude side lobes or apodization must be used. The inscription of two FBGs with optimal spectral characteristics will be addressed in future work and further experimental analysis will be performed. 

## Figures and Tables

**Table 1:** Parameter values for mathematical modeling.

Parameter	Value
$\upsilon_{n}$	1
${\bar{\delta n}}_{\mathrm{eff}}$	$1\times 10^{-4}$
$n_{\mathrm{eff}}$	1.458
$p_{e}$	$2.2\times 10^{-1}$
α	$5.5\times 10^{-7}$
ξ	$8.3\times 10^{-6}$
*r*	$8.9\times 10^{-1}$
*n*	1
θ	0 (rad)
*l*	$1.007\times \;10^{-7}$ (m)

**Table 2:** Parameter values for FBG tuning.

Parameter	Value
No. Iterations	100
No. Search agents	20
*b*	$5\times 10^{-4}$
$s_{\epsilon }$	1.2 (pm/μϵ)
$\epsilon_{max}$	700 (μϵ)
$s_{T}$	12 (pm/°C)
$T_{max}$	45 (°C)
$T_{min}$	15 (°C)
$\delta_{T}$	1 (°C)

## Data Availability

Not applicable.
